# Effects of Combined Vitamin K2 and Vitamin D3 Supplementation on Na[^18^F]F PET/MRI in Patients with Carotid Artery Disease: The INTRICATE Rationale and Trial Design

**DOI:** 10.3390/nu13030994

**Published:** 2021-03-19

**Authors:** Alexandru Florea, M. Eline Kooi, Werner Mess, Leon J. Schurgers, Jan Bucerius, Felix M. Mottaghy

**Affiliations:** 1 Department of Nuclear Medicine, University Hospital RWTH Aachen, 52074 Aachen, Germany; aflorea@ukaachen.de; 2 Department of Radiology and Nuclear Medicine, Maastricht University Medical Center, 6229HX Maastricht, The Netherlands; eline.kooi@mumc.nl (M.E.K.); jan.bucerius@med.uni-goettingen.de (J.B.); 3 School for Cardiovascular Diseases (CARIM), Maastricht University, 6229HX Maastricht, The Netherlands; l.schurgers@maastrichtuniversity.nl; 4 Department of Clinical Neurophysiology, Maastricht University Medical Center, 6229HX Maastricht, The Netherlands; werner.mess@mumc.nl; 5 Department of Biochemistry, Maastricht University, 6229HX Maastricht, The Netherlands; 6 Institute of Experimental Medicine and Systems Biology, RWTH Aachen University, 52074 Aachen, Germany; 7 Department of Nuclear Medicine, University of Göttingen, 37075 Göttingen, Germany

**Keywords:** cardiovascular diseases, positron emission tomography, magnetic resonance imaging, sodium fluoride, vitamin K, vitamin D, vascular calcification, micro-calcification

## Abstract

INTRICATE is a prospective double-blind placebo-controlled feasibility study, assessing the influence of combined vitamin K2 and vitamin D3 supplementation on micro-calcification in carotid artery disease as imaged by hybrid Sodium [^18^F]Fluoride (Na[^18^F]F) positron emission tomography (PET)/ magnetic resonance imaging (MRI). Arterial calcification is an actively regulated process and results from the imbalance between calcification promoting and inhibiting factors. Considering the recent advancements in medical imaging, ultrasound (US), PET/MRI, and computed tomography (CT) can be used for the selection and stratification of patients with atherosclerosis. Fifty-two subjects with asymptomatic carotid artery disease on at least one side of the neck will be included in the study. At baseline, an Na[^18^F]F PET/MRI and CT examination will be performed. Afterwards, subjects will be randomized (1:1) to a vitamin K (400 µg MK-7/day) and vitamin D3 (80 µg/day) or to placebo. At the 3-month follow-up, subjects will undergo a second Na[^18^F]F PET/MRI and CT scan. The primary endpoint is the change in Na[^18^F]F PET/MRI (baseline vs. after 3 months) in the treatment group as compared to the placebo arm. Secondary endpoints are changes in plaque composition and in blood-biomarkers. The INTRICATE trial bears the potential to open novel avenues for future large scale randomized controlled trials to intervene in the plaque development and micro-calcification progression.

## 1. Introduction

Arterial calcification is an active process and results from the imbalance between calcification promoting and inhibiting factors [1]. Extracellular matrix calcification is initiated by matrix vesicles, secreted by osteoblasts or osteoblast-like cells (i.e., vascular smooth muscle cells that acquired the pathological osteogenic phenotype [2]), inducing the nucleation of calcium phosphate crystals (i.e., hydroxyapatite) [3]. Accumulation of calcium and phosphate ions into vesicles will initiate crystal formation [3]. Next, hydroxyapatite crystals will elongate radially, penetrate the vesicle membrane, and will form calcifying nodules with neighboring matrix vesicles [3]. The subsequent growth of the calcifying nodule seems to be regulated by the availability of organic compounds (e.g., matrix Gla protein, MGP) [3].

Considering the recent advancements in medical imaging, ultrasound (US), magnetic resonance imaging (MRI), computed tomography (CT), as well as positron emission tomography (PET) with different (new) PET-tracers can be used for the selection and stratification of patients with atherosclerosis [4,5,6,7]. In a recent review, the superiority of sodium [^18^F]Fluoride (Na[^18^F]F) PET over other modalities to correctly identify micro-calcified plaques has been elaborated [8]. Na[^18^F]F PET is considered to be the only available clinical tool that can non-invasively detect (potentially) vulnerable micro-calcified plaques more sensitively than CT [8,9].

In the last two decades, a series of proteins that can bind calcium ions have been discovered and most of them share a common feature, the γ-carboxyglutamic acid (Gla) rich domain. As a result that Gla-residues are biologically converted from protein-bound glutamic acid residues by an enzyme which uses vitamin K as a cofactor, all these proteins are called vitamin K dependent proteins. MGP [10] is one of the most studied vitamin K dependent proteins that has proven to have a role in the protection against ectopic calcification [11,12].

MGP is able to avidly bind the calcium from hydroxyapatite crystals [13] and therefore to block the growth of calcifying nodules. In order to become biologically active, MGP requires two post-translational modifications: γ-carboxylation and phosphorylation.

MGP can be found in the blood stream and there are numerous human and animal studies in which MGP has been used as a marker for different inflammatory diseases that also involve calcification (e.g., coronary artery disease, type 2 diabetes, chronic kidney disease) [14,15,16]. High plasma levels of dephosphorylated uncarboxylated MGP (dp-ucMGP) correlate with an increased cardiovascular risk in type 2 diabetes patients [17]. Moreover, dp-ucMGP inversely correlates with vascular calcification levels of patients suffering from coronary artery disease [18], type 2 diabetes [19], and end stage kidney disease [20]. One study did not measure the inactive form, but the total concentration of MGP and found a significant reduction in MGP synthesis in patients with aortic valve calcification [21] and chronic kidney disease [22]. Moreover, low plasma levels of dp-ucMGP are associated with an increased all-cause and cardiovascular mortality in the general European population [23].

Therefore, dp-ucMGP might be used as a marker for vitamin K status and in extension for vascular health.

In a recent review paper, we have presented emerging data, which suggests vitamin K—especially Menaquinone-7 (MK-7)—as a cost-effective method of delaying progression of vascular calcification [8]. Although these studies support the idea that MK-7 protects against vascular calcification, there have been no controlled trials in humans assessing the effects of MK-7 supplementation on arterial calcification in the context of atherosclerosis.

Experimental studies suggest positive effects of vitamin D on vitamin K-dependent metabolism [24,25,26,27]. In mice, a diet high in active vitamin D for 20 weeks induced higher amounts of uncarboxylated (inactive) MGP, reflecting low vitamin K status and renal calcification [24]. Indeed, large randomized clinical trials failed to show a positive effect of vitamin D3 supplementation in the prevention of cardiovascular disease [28,29]. Vitamin K supplementation can overcome this untoward effect of excess vitamin D on calcification as demonstrated by lower calcium and phosphorus content in aorta and kidney [27]. The MGP-gene promoter contains a vitamin D response element, capable of a two to threefold enhanced MGP expression after vitamin D binding [25,30]. The upregulation of MGP due to vitamin D needs vitamin K to ensure full activation of MGP for optimal functioning. This implies that the combination of both vitamin K and vitamin D could provide enhanced protection against progressive vascular calcification, cardiovascular disease, and mortality [31].

It is for these above-mentioned reasons that we intend to use Na[^18^F]F PET/MRI in the proposed feasibility study, which will assess the influence of MK-7 and vitamin D3 supplementation on the development of arterial micro-calcification in the context of atherosclerosis. The primary objective of this study is to assess the combination of vitamin K and vitamin D on the pathophysiological process of arterial micro-calcification in carotid artery disease via hybrid Na[^18^F]F PET/MRI. Furthermore, the longitudinal change of micro-calcifications under MK-7 and D3 supplementation in the coronary arteries will be assessed by means of a coronary artery calcification (CAC) scan. The aforementioned scans will be compared between baseline and end of the study in a group receiving the vitamin supplementation as compared to placebo. In addition, dp-ucMGP will be measured in order to determine whether this biomarker can be used to assess the degree of calcification in the arterial wall of patients with coronary and carotid artery disease.

## 2. Study Design

The INTRICATE trial (acronym for the “Effects of combined vitamin K2 and vitamin D3 supplementation on Na[^18^F]F PET/MRI in patients with carotid artery disease” trial) is a double-blind randomized, placebo-controlled feasibility study, performed at the Maastricht University Medical Center, with an end of the study visit after 3 months. The research file of this trial, including the study protocol, was approved by the accredited local Medical Ethics Committee of the Academic Hospital Maastricht and University of Maastricht in the Netherlands (i.e., METC azM/UM) under the file number NL69450.068.19/METC300103 and is being conducted according to the principles of the Declaration of Helsinki. The INTRICATE trial is also registered at clinicaltrials.gov under NCT04010578. All subjects give their written informed consent for inclusion before they participate in the study. The informed consent is being received in writing, in the native language of the subjects (i.e., Dutch) and is available in the Supplementary Materials.

Subjects with asymptomatic carotid artery disease (i.e., on the common or the internal carotid) on at least one side with a degree of stenosis > 25% (according to ECST criteria) as detected by means of B-mode US and who meet the inclusion criteria will be recruited. After providing informed consent, they will be randomized in two groups:

1. the first group will receive a daily dose of 400 µg MK-7 and 80 µg D3 and
2. the second (i.e., control) group will receive a placebo.

Both, the investigators as well as the patients will be blinded regarding the treatment used.

At baseline, a Na[^18^F]F PET/MRI, a CAC scan, and an US examination of the neck vessels will be performed; moreover, samples of venous blood will be taken. Subjects will be asked for a follow-up evaluation after 3 months of supplementation, when Na[^18^F]F PET/MRI, CAC scan, and US scan will be repeated, in order to measure the degree of vascular calcifications and the changes in time of atherosclerosis. Additionally, venous blood samples will be obtained by standard venous puncture for routine biochemical, hematological, and serum biomarker assessments.

## 3. Study Population

Patients with asymptomatic carotid artery disease on one side, who meet the inclusion criteria and who provide a signed informed consent are being invited in this study.

### 3.1. Inclusion Criteria

In order to be eligible to participate in this study, a subject must meet all following criteria:

- Asymptomatic carotid artery disease on at least one side with a degree of stenosis > 25% (according to the ECST criteria). If the patient has a symptomatic carotid artery disease on the contra-lateral side, he/she will still be included in the study, if intensified medical treatment for this symptomatic stenosis (e.g., statins, antiplatelet medication) was started ≥6 month before inclusion of the patient. This protocol was chosen in order to assure a stable situation on the plaque(s), which avoids an overspill from this medication on the assumed effects of the supplementation.
- Age older than 18 years.
- Signed informed consent provided.

### 3.2. Exclusion Criteria

A potential subject who meets any of the following criteria will be excluded from participation in this study:

- Antiplatelet or cholesterol lowering medication started within the past 6 months.
- Chronic or paroxysmal atrial fibrillation.
- Already performed or scheduled coronary or carotid revascularization procedure (e.g., stent implantation, coronary artery bypass graft, balloon-dilatation, endarterectomy, angioplasty).
- History of myocardial infarction or stroke.
- Malignant disease (except for treated basal-cell or squamous cell carcinoma).
- Use of vitamin K antagonists treatment.
- A life-expectancy < 1 year.
- Claustrophobia.
- Presence of a pacemaker, intra-cardiac defibrillator, or metallic implant (e.g., vascular clip, neuro-stimulator, cochlear implant, metal splinter in the eye).
- Body weight > 130 kg or body habitus that does not fit into the gantry.
- Pregnancy or wish to become pregnant in the near future.
- Breast feeding.
- (History of) metabolic or gastrointestinal disease.
- Use of vitamin K or D containing supplements or vitamin K-rich foods (e.g., fermented soya).
- Chronic inflammatory disease.
- Systemic treatment or topical treatment likely to interfere with evaluation of the study parameters.
- Corticoid treatment.
- Participation in a clinical study within one month before enrolment in the current study.

## 4. Study Objectives and Statistical Analyses Plan

The objective of this study is to assess the influence of MK-7 and vitamin D3 on the pathophysiological process of arterial micro-calcification in carotid artery disease as assessed by hybrid Na[^18^F]F PET/MRI. Furthermore, the longitudinal change of micro-calcifications and plaque composition and volume under this supplementation in coronary artery disease patients will be assessed by means of MRI and CAC scan. The aforementioned scans will be compared between baseline and the end of the study visit in a group receiving vitamin K supplementation as compared to the placebo group. In addition, dp-ucMGP will be measured, in order to determine whether this biomarker can be used to assess the degree of calcification in the arterial wall of patients with coronary and carotid artery disease. Finally, blood samples will be stored in the Biobank, creating the opportunity to investigate new biomarkers regarding vitamins K and D status in the future (e.g., their influence on osteocalcin or alkaline phosphatase).

### 4.1. Primary Objective

We investigated whether MK-7 and vitamin D3 supplementation can diminish, halt, or even reverse the development of arterial micro-calcification in the carotid arteries in the context of atherosclerosis as detected by Na[^18^F]F PET/MRI compared to placebo after 3 months.

### 4.2. Secondary Objectives

Secondary objectives of the trial include assessing whether:

- the vitamin supplementation can diminish, halt, or even reverse the development of arterial micro-calcification in the coronary arteries in the context of atherosclerosis as detected by CAC score compared to placebo after 3 months;
- the primary parameter correlates or is able to predict the results of the CAC score;
- the vitamin supplementation can influence MRI parameters such as normalized wall index (i.e., measurement of plaque burden), intra-plaque hemorrhage volume, lipid-rich necrotic core volume, and fibrous cap status;
- the vitamin supplementation can influence US after 3 months of supplementation;
- Na[^18^F]F uptake in the carotids is associated with US at the end of the study visit;
- the vitamin supplementation is influencing plasma levels of dp-ucMGP after 3 months, in comparison with a placebo, in order to select a possible peripheral marker for vitamin K-status;
- baseline plasma levels of dp-ucMGP can be reliable biomarkers of plaque vulnerability and of its calcified state;
- there is a correlation between serum MK-7 concentrations and the values of the prothrombin time (PT) or the international normalized ratio (INR).

### 4.3. Statistical Analyses Plan

Our null hypotheses states that MK-7 and vitamin D3 do not influence the change (i.e., progression) of micro-calcifications in 3 months of supplementation when compared to a placebo; there will be no difference in the degree of calcification between the MK7+D3 group vs. the placebo at the end of the study. Our alternative hypothesis states that MK-7 and vitamin D3 have a greater effect on the change of micro-calcification in 3 months of supplementation compared to placebo.

The descriptive statistics for each variable at baseline will be presented for both groups combined and individually (i.e., MK7+D3 and placebo) for each of the following parameters: mean, standard deviation of the variable, standard error of the mean, range (minimum-maximum), median, quartiles, and confidence intervals where necessary. Descriptive statistics for variables at the end of the study will be presented separate for both groups (i.e., MK7+D3 and placebo).

Both intention-to-treat and per protocol analysis will be performed. For the intention-to-treat analysis, all randomized subjects (with data available from the end of the study visit) will be included. In the per protocol analysis, only subjects who fully complied with the prescribed supplementation will be included.

The main outcome parameter (i.e., the longitudinal change time of micro-calcification as measured by the Na[^18^F]F uptake via hybrid PET/MRI) will be presented as a continuous variable. It will be expressed as a mean difference (i.e., the standard uptake value and target-to-background radio of Na[^18^F]F at the end of the study visit minus Na[^18^F]F maximal uptake at baseline). To investigate the existence of significant predictor(s) for the main outcome parameter (i.e., the longitudinal change of micro-calcification), univariate analysis will be used. If the significance level (i.e., α) will be between 5% and 25% at the univariate *t*-test hypothesis testing (i.e., *p* > 0.05 and *p* < 0.25), a multivariate analysis will be used, in order to investigate a possible association or interaction between variables.

In order to decide to either reject or accept our null hypothesis, a two-sided confidence interval using 95% confidence will be calculated and reported. A two-sided hypothesis test using a significance level (i.e., α) of 5% will also be reported.

The CAC score and the US data will be presented as continuous variables. Their longitudinal change will be also expressed as a mean difference (i.e., value at the end of the study visit minus value at baseline) and will be presented as continuous variables.

For the continuous data including age, blood pressure, body-mass index, lipid-rich necrotic volume, normalized wall index, dp-ucMGP, and other laboratory tests, the distribution of variables will be analyzed (verification of normality of distribution for all subjects) by histograms. To investigate the existence of significant predictor(s) for the secondary outcome parameters, univariate analysis will be used. If the significance level (i.e., α) will be between 5% and 25% at the univariate t-test hypothesis testing (i.e., *p* > 0.05 and *p* < 0.25), a multivariate analysis will be used, in order to investigate a possible association or interaction between variables. If variables display no normal distribution, data will be transformed using appropriate transformations. If transformation is not possible, this data is analyzed by non-parametric statistical tests (i.e., Chi-square test).

Baseline parameters will be presented: sex, age, bodyweight, length, body-mass index, medical history, medication, family history, and cardiovascular risk factors.

Health status and use/changes of medication will be verified during the baseline visit and at the end of the study visit.

## 5. Study Procedures

An outline in chronological order, with all the procedures that will be received by the subjects, is available in Table 1.

### 5.1. Hospital Visit

At baseline visit, a complete medical interview and physical examination will be performed with emphasis on the detection of signs of cardiovascular disease (i.e., coronary and carotid artery disease). Furthermore, the height, weight, and waist-hip circumference will be measured.

Both at baseline and end of the study visits arterial blood pressure will be measured on both arms with an electronic blood-pressure measurement device. Before measurement, the subject will be seated for at least 5 min. Moreover, an ECG will be acquired.

### 5.2. Na[^18^F]F PET/MRI

The PET/MRI will be performed using a hybrid PET/MRI scanner (Siemens Biograph mMR, Siemens, Forchheim, Germany). During the PET scan (on hybrid PET/MRI scanner), MRI images will be concomitantly acquired to the PET images. A dedicated coil will be used for imaging of the carotid bifurcation. This coil allows for sub-millimeter resolution imaging of the lumen, vessel walls, and atherosclerotic plaques.

As Na[^18^F]F is eliminated by the kidneys, it will accumulate in the urinary bladder. In order to reduce the radiation exposure to the entire urinary tract, subjects will be asked to drink 0.5 L water in the hour before arrival at the hospital; for Na[^18^F]F, there is no fasting required. Upon arrival, study participants will be asked to drink an additional 0.5 L water and the procedure will be explained once more. Afterwards, 185 MBq of Na[^18^F]F will be injected via a venipuncture of the arm. Subjects will be asked to empty their bladder right before the start of the scan (i.e., 1 h post injection). The patient will be in supine position during scanning. The total scanning procedure will take about 1 h.

First, the carotid bifurcation will be identified by means of MR angiography without contrast agent enhancement. Subsequently, transverse images will be obtained around the carotid bifurcation, so that the complete plaque will be imaged for all patients. A multi-sequence MRI protocol will be used, including a dark blood T1 and a bright blood sequence to assess the juxtaluminal calcifications, a hyper T1 weighted sequence for intra-plaque hemorrhage, and a post-contrast dark blood T1 weighted sequence to assess the lipid-rich necrotic core and fibrous cap status. Concomitantly with the MRI, a static PET scan will be performed. The total PET/MRI examination time will take approximately 1 h.

Images derived from the PET/MRI will be used to determine the uptake of Na[^18^F]F, which is well known to sensitively reflect the degree of vascular calcification. Secondly, the MRI images will serve for evaluation of the carotid artery morphology. For this purpose, a contrast agent (i.e., Gadobutrol) is injected (dose 0.1 mmol/kg of body weight). This MRI contrast agent will be injected according to the standard medical practice of the Maastricht University Medical Center as described within the respective ODIN protocol.

#### 5.2.1. PET Image Evaluation

A certified nuclear medicine physician will evaluate all the PET images, which are corrected for attenuation based on the MRI data, unaware of all clinical information, outcome of the extensive MRI analysis. A dedicated fusion software (Syngovia, Siemens) will be used to analyze the PET images. Mean and maximum standard uptake values (i.e., SUVs) will be normalized for blood tracer activity by dividing them by the mean standard uptake value of blood as measured in the internal jugular vein, the superior vena cava, or the right atrium depending on the vessel wall of interest.

#### 5.2.2. MRI Evaluation

The MRI reader will be trained using an independent training set. This training set is developed to be able to identify all different plaque characteristics, which are scored during this study. The reader will have to independently score images in this independent training set that has been previously scored by a highly experienced reader, to determine the inter-observer agreement. If the inter-observer agreement is not good or not very good, then the reader will be further trained until he performs excellent reading of the training set (i.e., good or very good inter-observer agreement with the highly experienced reader). The MRI reader, blinded to the results from the other modalities and clinical data, will independently score carotid plaque characteristics on the MR Images (vessel wall and lumen area, size of lipid-rich necrotic core, and size of calcifications, fibrous cap status, and size of intra-plaque hemorrhage). All data will be entered in a common database and analyzed by the investigator, supervised by a statistician.

### 5.3. CAC Scan

Non-contrast enhanced CT (Somatom FORCE, Siemens Healthcare), which will determine the CAC score, will be obtained at baseline and at the end of the study visits. The scans will be made using standard procedure.

The patient will be in supine position with his/her arms raised above his/her head during scanning. In addition, he/she is required to hold his/her breath for several seconds. The total procedure will take about 2–3 min. The procedure described above is the standard scanning-method in this hospital. The CAC score will be determined by performing a non-contrast enhanced high pitch scan.

All images will be assessed in consensus between an experienced radiologist and cardiologist. In case of disagreement, consensus will be reached by reviewing findings jointly.

As with the PET and MRI images, all CT images will also be examined by dedicated radiologists for incidental findings, which then will be reported to the general practitioner of the patients. Patients who do not want to be informed about additional findings are not able to participate in this study.

### 5.4. US Examination

The US examination is performed with the Art-Lab system (Esaote/Pie Medical, Maastricht, The Netherlands). With the subject lying supine, both common carotid arteries are identified on a longitudinal US image. On both walls of the common carotid arteries, a double line pattern can be observed, consisting of the edges of the lumen-intima-transition and media-adventitia-transition. On the far wall of the common carotid artery, the intima-media thickness is measured as the distance between these two lines in micrometers.

Additionally, the following parameters are measured:

- gray-scale median of the intima-media thickness;
- gray-scale median of the plaque;
- adventitia-adventitia diameter;
- intima-intima diameter;
- plaque echolucency.

The total duration of this investigation is approximately 15 min.

### 5.5. Laboratory Assessments

At baseline and at the end of the study visits, blood-samples (after an overnight fast) will be obtained for the measurement of several different cardiovascular parameters and markers of calcification and vitamin K status. In addition, 7 mL will be stored for future research. In total, almost 25 mL of blood will be obtained per study-visit for blood analysis. Therefore, almost 50 mL per subject for the entire project.

Blood samples will be processed by the Biobank Maastricht within 1 h and stored at −80 °C until analysis. We will investigate whether an association between biomarkers and calcification exists in an experimental setting. Therefore, we do not intend to inform the subjects of the blood results.

The routine assessments will be:

- total cholesterol;
- LDL-cholesterol;
- HDL-cholesterol;
- triglycerides;
- creatinine;
- glucose;
- albumin;
- parathyroid hormone;
- calcium;
- phosphate;
- coagulation function (e.g., PT, INR).

Specific laboratory assessments will consist in measuring the dp-ucMGP blood level.

## 6. Randomization, Blinding, and Treatment Allocation

A custom-made software with minimization as a randomization method (i.e., operated by the researcher) will randomize consecutive patients in the two arms, using the carotid stenosis as a stratification factor. The software will stratify the subjects according to the initial carotid stenosis into one of the following groups: stenosis 25–50% or stenosis > 50%. This software will assign subjects with a specific randomization number. This randomization number will be used by NattoPharma (Oslo, Norway) for coding and labelling of the capsule bottles. The list with all the randomization number will be stored in a secure location and will not be accessible for the investigators during this study.

The study code will be de-blinded if a serious adverse event occurs in an individual subject.

## 7. Investigational Product

Subjects in the intervention-group will receive a daily dose of 400 µg MK-7 and 80 µg vitamin D3. Each intervention capsule contains Cholecalciferol (per capsule: 1.6 mg of 1,000,000 UI/g; 1 UI of vitamin D = 0.025 μg) and MenaQ7 K2 oil (per capsule: 133.33 mg of 1500 ppm vitamin K).

MenaQ7 K2 oil is a synthetic MK-7 oil produced by NattoPharma (Oslo, Norway). Euro-Pharma Alliance Sp. zo.o. (Rzeplin, Poland) uses this oil to manufacture the capsules, which are later marketed by TG Montgomery AS (Oslo, Norway). TG Montgomery AS also distributes them as a food supplement on the Norwegian over-the-counter market. MenaQ7 is Generally Recognized as Safe (i.e., GRAS) by the U.S. Food and Drug Administration (i.e., FDA).

Both MK-7 and vitamin D3 are registered as food-supplements. MK-7 is well tolerated and does not cause a state of hypercoagulability [32]. There are no reported negative side effects associated with the use of MK-7 or vitamin D3, at the concentrations used in this study.

Subjects in the placebo-group will receive a capsule that is identical to the MK7+D3 one, but that does not contain any active substances. In all other aspects (e.g., shape, taste, weight, additives), this capsule is similar to the MK7+D3 one. The shell of both type of capsules is the same and compared to the MK-7+D3 ones, the placebos only contain bulking agent (the same one, i.e., linseed oil) in a higher amount, so that both capsules have the same weight. There are no reported negative side effects with the use of this placebo.

Both capsules are provided by TG Montgomery as a complete end-product ready to use and packaged separately for each participant in bottles. They are stored according to the manufacturer recommendation, which has already tested the shelf life and recommended a maximum storage time. This information will also be given to the subjects and they may request at any time a bottle replacement, in case of improper storage or passing the recommended maximum storage time.

## 8. Discussion

Both Phylloquinone (i.e., vitamin K1) and Menaquinones (i.e., vitamin K2) consist of a naphthoquinone-ring structure and an aliphatic sidechain [33]. The bioactive part of vitamin K is the naphthoquinone-ring structure [33]. MK-4 has already been proposed for the treatment of osteoporosis in Japan [32]; however, the same supplementation failed to show positive effects on coronary artery calcification and on vascular stiffness [34]. The selection of MK-7 over other types of vitamin K was done based on its already proven positive effects on arterial stiffness and on the risk of coronary heart disease [35,36].

The INTRICATE trial is a proof-of-concept study, in which the effects of MK-7 and vitamin D3 on vascular calcification will be assessed via Na[^18^F]F PET. In a randomized double-blind placebo-controlled trial of 340 adults, vitamin D3 alone showed a reduction in pulse wave velocity, an indirect marked of arterial stiffness [37].

However, there are also other vitamins (i.e., B12, C, and E) being studied in the context of vascular calcification, which showed promising results [38]. In another randomized controlled trial, co-administration of intravenous vitamin B12 and oral folate in hemodialysis patients showed improved effects on arterial stiffness compared to folate single treatment [39]. On the other hand, vitamin A failed in several clinical trials to show a protection against cardiovascular diseases [38,40].

There are many other intervention treatments which may be assessed with Na[^18^F]F PET. Taking into consideration the burden of the subjects, who will be included in the INTRICATE trial, a safe and cost-effective treatment was used to show the treatment monitorization potential of this imaging technique in cardiovascular diseases [8].

## 9. Summary

The INTRICATE study is a proof-of-concept trial that will provide us with information on micro-calcification development in the context of atherosclerosis in the carotid arteries and the potential effect of supplementation with vitamin K (i.e., MK-7) and vitamin D3. This trial bears the potential to open novel avenues for future large scale randomized controlled trials to intervene in the plaque development and micro-calcification progression.

## Figures and Tables

**Table 1:** Outline of visit procedures.

Study Procedure	Recruitment (*t* ≈ −7 Days)	Baseline Visit (*t* = 0)	End of the Study Visit (*t* ≈ 3 Months)
Informed consent	X ^1^		
Hospital visit	X	X ^1^	X ^1^
demographic factors	X		
medical history	X		
current medication	X	X ^1^	X ^1^
physical examination	X	X ^1^	X ^1^
blood pressure	X		X
ECG	X		X
Blood sampling and laboratory assessments		X ^1^	X ^1^
US examination		X ^1^	X ^1^
Na[^18^F]F PET/MRI		X ^1^	X ^1^
CAC scan		X ^1^	X ^1^
Randomization		X ^1^	
Drug distribution		X ^1^	
Drug count			X ^1^

^1^ Different from routine clinical care.

## Data Availability

Data sharing is not applicable. No new data were created or analyzed in this study.

## Supplementary Materials

The following are available online at https://www.mdpi.com/2072-6643/13/3/994/s1, Text S1: Informed consent.
