# Supplementary material for: Effects of Combined Vitamin K2 and Vitamin D3 Supplementation on Na[18F]F PET/MRI in Patients with Carotid Artery Disease: The INTRICATE Rationale and Trial Design
Source: Nutrients. 2021 Mar 19;13(3):994. doi: 10.3390/nu13030994 (PMC8003489; doi:10.3390/nu13030994)
Supplement: Supplementary file 1 [file nutrients-13-00994-s001.pdf]

Toestemmingsformulier proefpersoon

- Ik heb de informatiebrief gelezen. Ook kon ik vragen stellen. Mijn vragen zijn voldoende beantwoord. Ik had genoeg tijd om te beslissen of ik meedoe.
- Ik weet dat meedoen vrijwillig is. Ook weet ik dat ik op ieder moment kan beslissen om toch niet mee te doen of te stoppen met het onderzoek. Daarvoor hoef ik geen reden te geven.
- Ik geef toestemming voor het informeren van mijn huisarts en specialist die mij behandelt dat ik meedoe aan dit onderzoek.
- Ik geef toestemming voor het verzamelen en gebruiken van mijn gegevens en bloedmonsters voor de beantwoording van de onderzoeksvraag in dit onderzoek.
- Ik weet dat voor de controle van het onderzoek sommige mensen toegang tot al mijn gegevens kunnen krijgen. Die mensen staan vermeld in deze informatiebrief. Ik geef toestemming voor die inzage door deze personen.
- Ik geef toestemming voor het informeren van mijn huisarts en/of behandelend specialist van onverwachte bevindingen die van belang (kunnen) zijn voor mijn gezondheid.
- Ik weet dat ik niet zwanger mag worden tijdens het onderzoek.
- De onderzoeker heeft de voor mij meest geschikte anticonceptie met mij besproken.
- Ik geef □ **wel**
□ **geen** toestemming om mijn persoonsgegevens langer te bewaren en te gebruiken voor toekomstig onderzoek op het gebied van halsslagaderziekte.
- Ik geef □ **wel**
□ **geen** toestemming om mijn lichaamsmateriaal na dit onderzoek te bewaren en om dit later nog voor verder onderzoek op het gebied van halsslagaderziekte te gebruiken, zoals in de informatiebrief staat.
- Ik geef □ **wel**
□ **geen** toestemming om mij na dit onderzoek opnieuw te benaderen voor een vervolgonderzoek.
- Ik wil □ **wel**
□ **niet** geïnformeerd worden over welke behandeling ik heb gehad/in welke groep ik zat.

- Ik wil meedoen aan dit onderzoek.

Naam proefpersoon: ____
Handtekening: Datum: ____ / ____ / ______

____

Ik verklaar dat ik deze proefpersoon volledig heb geïnformeerd over het genoemde onderzoek.

Als er tijdens het onderzoek informatie bekend wordt die de toestemming van de proefpersoon zou kunnen beïnvloeden, dan breng ik hem/haar daarvan tijdig op de hoogte.

Naam onderzoeker (of diens vertegenwoordiger): ________________________________
Handtekening: Datum: ____ / ____ / ______

____

De proefpersoon krijgt een volledige informatiebrief mee, samen met een getekende versie van het toestemmingsformulier.
